# Acute limb ischemia in a cancer patient has high morbidity, high mortality, and atypical presentation: a tertiary cancer center’s retrospective study

**DOI:** 10.1186/s12885-021-08659-x

**Published:** 2021-08-13

**Authors:** Yolanda Bryce, Amoateng Emmanuel, Christopher Agrusa, Etay Ziv, Christopher Harnain, Samantha Huq, Ernesto Santos Martin

**Affiliations:** 1 Memorial Sloan Kettering Cancer Center, 1275 York Avenue, New York, NY 10065 USA; 2 City University of NY (CUNY) School of Medicine, 160 Convent Avenue, Convent Ave, New York, NY 10031 USA; 3 New York Presbyterian Hospital Weill Cornell Medical Center, 525 East 68th Street Starr 8, New York, NY 10065 USA

**Keywords:** Acute limb ischemia, cancer patient, Atypical presentation, Peripheral arterial disease, embolus

## Abstract

**Background:**

Acute Limb Ischemia (ALI) carries a high morbidity and mortality rate that is compounded in the cancer patient. Though it is a relatively uncommon event, it is of extremely high adverse impact and carries poor awareness among clinicians.

**Methods:**

Retrospective review of electronic medical records was performed of cancer patients presenting with acute limb ischemia (ALI) to the tertiary cancer center’s urgent care center or as inpatient between January 1, 2014 and January 1, 2020.

**Results:**

Out of the 29 cancer patients with ALI, 12 (41%) died within 3 month and 9 (31%) patients died within 1 months of ALI diagnosis. 65% had long term adverse outcome after ALI – 31% with death in 1 month, 2 (7%) with an amputation, 5 (17%) with lifestyle-limiting claudication, and 3 (10%) with subsequent wound ulceration or gangrene. Patients not eligible for standard of care (12 patients, 41%) (RR 2.33 95% CI [1.27–4.27], *p* <  0.01) and heparin administration ≥6 h from presentation (19 patients, 65%) (RR 2.81 [1.07–7.38], *p* = 0.04) were at increased risk of adverse outcome. Atypical/confounded presentation of ALI (13 patients, 45%) (RR 1.84 95% CI [1.03–3.29], *p* = 0.04), pulse exam not documented (12 patients, 41.4%) (RR 1.95 [95% CI [1.14–3.32], *p* = 0.01), and patients with services other than a vascular specialist initially consulted (8 patients, 27.6%) (RR 1.91 95% CI [1.27–2.87], *p* <  0.01) were significant risk factors for heparin administered ≥6 h from presentation.

**Conclusions:**

ALI is devastating in cancer patients, with a high number presenting with atypical/confounded signs and symptoms which delays treatment. Heparin administered ≥6 h from presentation is associated with adverse outcome.

## Background

Acute Limb Ischemia (ALI) is caused by sudden interruption of arterial blood flow to a limb (most often the lower extremity) threatening the viability of the limb, defined as acute (< 2 week), severe hypoperfusion of the limb characterized by pain, pallor, pulselessness, poikilothermia (cold), paresthesias, and paralysis [1]. It is usually caused by peripheral arterial disease (PAD) with in situ thrombosis or embolus from a cardiac source due to arrythmias like atrial fibrillation [1, 2]. The incidence is as high as 22–26 per 100,000 per year and carries a major amputation rate of 15–25% and mortality rate of 15–20% within the first year after the ALI event [1]. In the cancer patient, is a relatively uncommon event compared to the general population, but it is of extremely high adverse impact and carries poor awareness among clinicians, especially cancer specialists [3]. The purpose of this study is to report a tertiary cancer center’s experience with ALI describing patient presentation, risk factors, and outcome associated with adverse outcome.

## Methods

This single-institution tertiary cancer center retrospective study was approved by the Institutional Review Board. A Database inquiry of inpatient and urgent care center admission utilizing ICD 9/10 codes of 174.2 Embolism and Thrombosis of the upper extremities, 174.3 Embolism and thrombosis of arteries of the lower extremities, 174.5 Embolism and thrombosis of iliac artery, 174.5 Embolism and thrombosis of iliac artery, and 444.22 Lower extremity embolism from years January 1, 2014 to January 1, 2020 was performed. One hundred sixty-two patients were obtained from the inquiry. Out of these patients, in reviewing records, 33 patients had the diagnosis of acute limb ischemia while others had venous obstruction or arterial embolization for hemorrhage or tumor control and were erroneously given the above ICD 9/10 codes. Four of the 33 patients were diagnosed and treated at an outside hospital and were excluded from the study. This left 29 patients for analysis. An electronic record review was done to assess demographics, cause of ALI, presentation, risk factors, treatment, time to anticoagulation and time to definitive treatment, type of treatment and outcome. Pulse examination (a critical component of the physical exam to assess for ALI) and whether other services were solicited before ALI was considered and referral made to a Vascular Specialist that treats ALI were also assessed.

Definitive treatment was described as final treatment decided upon – either watchful waiting as in patients who were not candidates for treatment, anticoagulation only therapy, endovascular procedure, or surgical procedure. Standard of care was defined as standard of care for ALI as recommended by 2016 AHA/ACC guidelines [4, 5].

Hypercoagulable state as cause of ALI was described as thrombosis ≥2 noncontiguous vascular beds in the setting of Disseminated Intravascular Coagulation (DIC) or progressed cancer with no other attributed cause for ALI. Embolic as cause of ALI was embolus due to cardiac or other sources. PAD as a cause of ALI were patients who had pre-existing PAD with ALI due to in situ arterial thrombosis. Iatrogenic as cause of ALI was due to a surgical or interventional radiology procedure that involved a vascular bed.

A typical ALI patient presentation was a patient presenting with limb pain plus or minus sensory loss, motor loss, limb coolness or color changes. Patients with other symptoms such as back pain, shortness of breath, nausea and vomiting, and rash that may distract from the diagnosis of ALI were considered an atypical or confounded ALI presentation.

Adverse outcome was classified as death within 1 month, amputation, lifestyle-limiting Rutherford 3 claudication, and chronic limb-threatening ischemia (CLTI). A table was constructed to reflect the clinical factors and outcomes between the no adverse outcome group and the adverse outcome group. Mann-Whitney U test and chi-squared test were used to identify any significant difference between both groups, as appropriate. Relative Risk analysis demonstrated any risk for death within 1 month and adverse outcome. Univariate and multivariate logistic regression modeling was used to assess prognostic variables for adverse outcome. Initiation of heparin ≥6 h from presentation to the UCC or as inpatient was assessed as one of the potential risk factors and prognostic variables. ≥ 6 h was used as an extension of the 2016 AHA/ACC guidelines, where the 6-h mark for ALI treatment is described [4, 5]. Statistical analyses were performed using MedCalc Statistical Software version 19.2.1 (MedCalc Software Ltd., Ostend, Belgium; https://www.medcalc.org; 2020). Significance was set at *p* <  0.05.

## Results

Twenty-nine patients met criteria for the study with mean age of 60 (± 14) years and median age of 61 years. 59% were men, 90% were white and 10% were Black. 83% were lower extremity ALI rather than upper extremity ALI. See Table 1. 8 (28%) were due to PAD with in situ thrombosis, 7 (24%) were due to embolus typically from a cardiac source due to an arrhythmia or heart failure but some of these patients also with underlying PAD increasing the propensity for ALI, 8 (28%) were iatrogenic from a procedure or surgery such as common femoral artery access for an angiogram or limb/vessel reconstruction after cancer resection, and 6 (21%) were due to a hypercoagulable state typically in the setting of DIC or progressed cancer with no other attributed cause for ALI. 18 (62%) patients had a threatened limb (Rutherford ALI 2a or 2b). Mean and median time to heparin drip was 17.5 h (range 0.4–153.6 h) and 8.6 h, respectively. Mean and median time to definitive treatment was 23.8 h (range 0.40–184.6 h) and 8.6 h, respectively. See Table 2.

**Table 1:** Baseline clinical factors in patients with ALI divided between the ALI group with adverse outcome and the ALI group with no adverse outcome

	Adverse Outcome	No Adverse Outcome	Total	***P***
Number of patients	19, 65.5%	10, 34.5%	29	N/A
Age years Mean (SD)	61 (13)	57 (20)	60 (14)	0.48
Age years (Median)	66	57	63	–
Male	10, 52.6%	7, 70.0%	17, 58.6%	0.37
White Race	16, 84.2%	10, 100.0%	26, 89.7%	0.19
Black Race	3, 15.8%	0, 0.0%	3, 10.3%	0.19
Genitourinary Cancer	3, 15.8%	3, 30.0%	6, 20.7%	0.38
Gastrointestinal Cancer	5, 26.3%	4, 40.0%	9, 31.0%	0.46
Gynecologic Cancer	1, 5.2%	0, 0.0%	1, 3.4%	0.47
Breast Cancer	1, 5.2%	0, 0.0%	1, 3.4%	0.47
Head and Neck Cancer	1, 5.2%	0, 0.0%	1, 3.4%	0.47
Thoracic (lung, mesothelioma)	3, 15.8%	0, 0.0%	3, 10.3%	0.19
Hematologic cancer	1, 5.2%	2, 20.0%	3, 10.3%	0.22
Bone/soft tissue sarcoma	3, 15.8%	0, 0.0%	3, 10.3%	0.19
Skin cancer	1, 5.2%	1, 10.0%	2, 6.9%	0.63
Stage 1	3, 15.8%	4, 40.0%	7, 24.1%	0.16
Stage 2	2, 10.5%	1, 10.0%	3, 10.3%	0.97
Stage 3	3, 15.8%	0, 0.0%	3, 10.3%	0.19
Stage 4	11, 57.9%	5, 50.0%	16, 55.2%	0.69
Active chemotherapy	7, 36.8%	2, 20.0%	9, 31.0%	0.36
Active prothrombotic chemotherapy	2, 10.5%	1, 10.0%	3, 10.3%	0.97
Active immunotherapy	4, 21.1%	0, 0.0%	4, 13.8%	0.12
Active radiation therapy	2, 10.5%	0, 0.0%	2, 6.9%	0.30
Clonal Hematopoiesis of Indeterminate Potential	1, 5.2%	1, 10.0%	2, 6.9%	0.63
Diabetes	6, 31.5%	4, 40.0%	10, 34.5%	0.65
Hypertension	11, 57.9%	7, 70.0%	18, 62.1%	0.53
Hyperlipidemia	6, 31.5%	4, 40.0%	10, 34.5%	0.65
Chronic kidney disease	5, 26.3%	2, 20.0%	7, 24.1%	0.71
Coronary artery disease	7, 36.8%	1, 10.0%	8, 27.6%	0.13
Cerebrovascular disease	0, 0.0%	0, 0.0%	0, 0.0%	–
Peripheral arterial disease	7, 36.8%	3, 30.0%	10, 34.5%	0.72
Obesity	5, 26.3%	3, 30.0%	8, 27.6%	0.84
Atrial fibrillation or other cardiac dysrhythmia	6, 31.5%	0, 0.0%	6, 20.7%	0.05
Smoking	7, 36.8%	5, 50.0%	12, 41.4%	0.48
Anti-platelet therapy	5, 26.3%	1, 10.0%	6, 20.7%	0.31
Statin therapy	2, 10.5%	2, 20.0%	4, 13.8%	0.49
Anticoagulation therapy	3, 15.8%	0, 0.0%	3, 10.3%	0.19
Anticoagulation indicated but held/not taken leading up to ALI	7, 36.8%	0, 0.0%	7, 24.1%	0.03

**Table 2:** ALI factors and outcome divided between the ALI group with adverse outcome and the ALI group with no adverse outcome

	Adverse Outcome	No Adverse Outcome	Total	***P***
Lower extremity ALI (verses upper extremity) n, %	16, 84.2%	8, 80.0%	24, 82.8%	0.78
Cause of ALI: Embolic n, %	6, 31.6%	1, 10.0%	7, 24.1%	0.20
Cause of ALI: Hypercoaguable State n, %	5, 26.3%	1, 10.0%	6, 20.7%	0.31
Cause of ALI: Iatrogenic n, %	3, 15.8%	5, 50.0%	8, 27.6%	0.05
Cause of ALI: PAD n, %	5, 26.3%	3, 30.0%	8, 27.6%	0.83
Threatened limb (Rutherford ALI 2a and 2b)	13, 68.4%	5, 50.0%	18, 62.1%	0.34
Atypical or confounded presentation, n, %	9, 47.4%	4, 40.0%	13, 44.8%	0.71
Pulses NOT documented	9, 47.4%	3, 30.0%	12, 41.4%	0.37
Patients with services other than vascular specialist consulted	7, 36.8%	1, 10.0%	8, 27.6%	0.13
Time to Heparin intravenous (initial treatment) Hours Mean (SD), range	23.6 (39.4), 1.0–153.6	5.9 (6.8), 0.4–20.4	17.5 (33.8), 0.4–153.6	0.18
Time to Heparin intravenous (initial treatment) Hours Median	10.1	2.4	8.6	–
Time to Definitive treatment Hours Mean (SD), range	23.0 (39.7), 1.0–153.6	25.1(55.9), 0.4–184.6	23.8 (45.8), 0.4–184.6	0.91
Time to Definitive treatment Hours Median	8.8	5.5	8.6	–
Heparin initiated ≥6 h from presentation	16, 84.2%	3, 30.0%	19, 65.5%	< 0.01
NOT eligible for ALI Standard of Care n, %	12, 63.2%	0, 0.0%	12, 41.4%	< 0.01
Death in follow up period n, %	12, 63.2%	1, 10.0%	13, 44.8%	0.01
Death in 3 months n, %	12, 63.2%	0, 0.0%	12, 41.4%	< 0.01
Death within 1 month n, %	9, 47.4%	N/A	9, 31.0%	N/A
ALI-related cause of death n, %	6, 31.6%	N/A	6, 46.1%	N/A
Cancer-related cause of death n, %	4, 21.1%	1, 10.0%	5, 38.4%	0.46
Cardiac arrest from Myocardial Infarction as cause of death n, %	2, 10.5%	0, 0.0%	2, 15.4%	0.30
Amputation n, %	2, 10.5%	N/A	2, 6.8%	N/A
Lifestyle-limiting claudication n, %	5, 26.3%	N/A	5, 17.2%	N/A
Chronic limb-threatening ischemia, n, %	3, 15.8%	N/A	3, 10.3%	N/A
Mean (SD) Follow up, months	6 (14)	24 (15)	12 (17)	< 0.01
Median Follow up, months	2	21	3	–

Mean follow up for the entire cohort was 12 months ±17 months, with median follow up of 3 months. In the follow up period 13 (45%) patients died, with 12 (41%) patients dying within 3 months of ALI diagnosis and 9 (31%) dying within 1 month of ALI diagnosis. Immediate causes of death were ALI-related (6, 46%), cancer-related (5, 38%), and cardiac arrest from a myocardial infarction (2, 15%). See Table 2.

Out of all 29 patients, only 9 (31%) were alive without adverse outcome in the follow up period. One patient died within the study period from cancer and did not have adverse ALI outcome. Those with classified adverse outcome (19, 65%) included – 9 (31%) dying within 30 days, 2 (7%) with an amputation, 5 (17%) with lifestyle-limiting claudication, and 3 (10%) with chronic limb-threatening ischemia with wound ulceration or gangrene (Table 2).

Between the group with adverse outcome and the group with no adverse outcome, there were statistically significant differences in number of patients with anticoagulation being held/not taken leading up to ALI (adverse outcome group 36.8% vs no adverse outcome group 0.0%, *p* = 0.03), heparin being administered ≥6 h from presentation (adverse outcome group 84.2% vs no adverse outcome group 30.0%, *p* <  0.01), number of patients not eligible for standard of care (adverse outcome group 63.2% vs. no adverse outcome group 0.0%, *p* < 0.01), death in the follow up period (adverse outcome group 63.2% vs. no adverse outcome 10.0%, *p* = 0.01), and death within 3 months (adverse outcome group 63.2% vs. no adverse outcome 0.0%, *p* < 0.01). Follow up was shorter in the adverse outcome group compared to the no adverse outcome group (6 months vs. 24 months, *p* < 0.01) consistent with the large amount of deaths in the adverse outcome group. All other factors (outside the definition of adverse outcome), including type of cancer, stage of cancer, cancer therapy, prothrombotic chemotherapy, comorbidities, the use of antiplatelet therapy, statin therapy, or anticoagulation were not statistically different between both groups. See Table 1.

Patients with hypercoagulable state were at increased risk of death within 1 month (RR 3.07 95% CI [1.17–8.01], *p* = 0.02). Patients NOT eligible for standard of care (RR 2.33 95% CI [1.27–4.27], *p* < 0.01) and heparin administration ≥6 h (RR 2.81 [1.07–7.38], *p* = 0.04) from presentation were at increased risk of adverse outcome. Patients not eligible for standard of care were patients with severe coagulopathy that could not be corrected such as in the case of DIC or ineligible for procedure or surgery due to severe end stage cancer and imminent death.

In univariate analysis, Heparin started ≥6 h from presentation was a predictor for adverse outcome with 12 times greater odds for adverse outcome than patients with heparin administered < 6 h (OR 12.44 [95% CI 2.00–77.60], *p* = 0.01) (Table 3). Atypical or confounded presentation (RR 1.84 95% CI [1.03–3.29], *p* = 0.04), pulse exam not documented (RR 1.95 [95% CI [1.14–3.32], *p* = 0.01), and services other than a vascular specialist consulted, (RR 1.91 95% CI [1.27–2.87], *p* < 0.01) were significant risk factors for heparin started ≥6 h from presentation (Table 4).

**Table 3:** Prognostic variables for adverse outcome in a logistic regression model

	OR	95% CI	***P*** (univariate)	***P*** (multivariate)
Cause of ALI: Embolus	4.15	0.42–40.66	0.22	
Cause of ALI: Hypercoaguable state	3.21	0.32–32.21	0.32	
Cause of ALI: Iatrogenic	0.19	0.03–1.08	0.06	
Cause of ALI: PAD	0.83	0.15–4.54	0.83	
Threatened Limb	2.167	0.45–10.44	0.34	
Not eligible for ALI standard of care	35.00	1.78–687.71	0.99	
Heparin administration ≥6 h	12.44	1.99–77.60	**0.01**	0.99

**Table 4:** Risk and significance of variables leading to administration of heparin ≥6 h from presentation

	Relative Risk	95% CI	***P***
Atypical or confounded presentation	1.84	1.03–3.29	**0.04**
Pulse not documented	1.95	1.14–3.32	**0.01**
Other services consulted	1.91	1.27–2.87	**< 0.01**

In the studied population, only 16 (55%) of patients had a typical presentation of ALI (Table 1). The other 13 (45%) patients presented with confounding signs and symptoms such as cancer-related pain such as back and radicular pain from retroperitoneal and spine metastatic involvement (4, one patient also with unrelated rash in the ipsilateral limb), post-surgical pain (2, one patient also with unrelated cellulitis in the ipsilateral limb), unrelated fever with pneumonia (1), unrelated severe shortness of breath from pulmonary embolus or CHF exacerbation (2), generalized weakness and dizziness due to advanced disease state/failure to thrive (1), unable to communicate symptoms due to unconsciousness (1), and small bowel obstruction with nausea/vomiting resulting in hypovolemia and arterial thrombosis in the setting of PAD (2).

12 (41%) patients did not have pulse exam documented at the time of presentation. In addition, 8 (28%) patients had referrals placed to other services such as Neurology, Pain, or Rheumatology Service before the diagnosis of ALI was considered and referral placed for a Vascular Specialist with the ability to treat ALI. See Table 2.

## Discussion

ALI is a devastating event with an extremely poor prognosis. It has a reported mortality rate of 15–20% within 1 year of the event, due to concomitant illness such as cardiovascular or cerebrovascular disease and ischemia–reperfusion injury [1, 4]. ALI may be compounded by cancer factors that worsen the outcome of ALI or prohibit standard of care for ALI. This study in cancer patients demonstrated a 3-month mortality rate of 41% and a 1-month mortality of 31% which is higher than reported values in the general population [1, 4]. ALI has a high mortality, but is also an extremely morbid disease with less than one-third of the cancer patients being alive and without long-term adverse outcome in the follow up period. 65% of the patients had an adverse outcome – death within 1 month (with hypercoaguable state being at increased risk for death within 1 month), amputation, lifestyle-limiting claudication, or chronic limb-threatening ischemia.

In this study, ALI was caused by PAD, embolus from a cardiac source, iatrogenic from procedure/surgery, and hypercoaguable state. Moreover, there were patients that had anticoagulation prescribed (typically in the setting of atrial fibrillation) but not taken either due to treatment related thrombocytopenia, pending cancer surgery/procedure, or noncompliance that resulted in ALI and was significantly overrepresented in the adverse outcome group.

41% of the patients did not have pulse exam documented in the setting of ALI and 28% had services consulted other than a vascular specialist at the time of ALI presentation. This may be because of lack of awareness among clinicians as has been repeatedly described in the literature when discussing findings associated with ALI in the general population [6–10]. However, this may also be because so many cancer patients do not present with the typical presentation that occurs in the general population. As in this study, pulse exam not documented, specialist other than a vascular specialist, and atypical presentation are factors that may overlap in patients, with one factor leading to another. For instance, a patient with atypical symptoms may not have a pulse exam and a vascular specialist may not be consulted. In this study, these factors led to administration of heparin 6 or more hours after presentation. Initiation of treatment ≥6 h from presentation was predictive of an adverse outcome. Therefore, the oncologist’s awareness and heightened suspicion for ALI may be helpful even in the setting of atypical/confounded presentation to mitigate delay so appropriate exam is performed and referral to a vascular specialist is made. Early diagnosis and prompt initiation of therapy are essential to avoiding an adverse outcome [11].

Prognosis of ALI patients is also dependent on appropriate treatment [12–16]. The Consensus by the American Heart Association/American College of Cardiologists recommends that marginally or immediately threatened limbs (Rutherford ALI 2a and 2b), should be revascularized emergently (within 6 h) and viable limbs (Rutherford ALI 1), should be revascularized on an urgent basis (within 6–24 h). The revascularization strategy ranges from catheter-directed thrombolysis to surgical thromboembolectomy. Systemic anticoagulation with intravenous heparin should be administered upon presentation to improve outcome [5]. However, 41% of the cancer patients in this study were not eligible to receive ALI standard of care due to poor procedure or anticoagulation candidacy due to cancer or cancer therapy complications. All patients not able to receive ALI standard of care were in the adverse outcome group. Patients not eligible for ALI standard of care had two times the risk of adverse outcome.

Limitations of this study include single-center small retrospective study with innate bias. Small sample size, due to the relatively uncommon event of ALI in cancer patients, was reflected in some of the wide confidence intervals. Prospectively studying cancer patients with ALI may more precisely elucidate reasons for poor outcome and improve outcome in this vulnerable population.

## Conclusions

ALI is a devastating event especially in a cancer population, carrying an extremely high morbidity and mortality, with less than one-third of the cancer patients in this study alive and without adverse outcome in the follow up period. 45% of the cancer patients had an atypical or confounded ALI presentation delaying diagnosis. Initiation of heparin ≥6 h from presentation was predictive of adverse outcome. Moreover, 41% of the cancer patients in this study were not able to receive standard of care for ALI with increased risk of adverse outcome.

## Data Availability

The minimal dataset that is necessary to interpret, replicate, and build upon findings are available from the corresponding author on reasonable request.

## Abbreviations

ALI: Acute Limb Ischemia
PAD: Peripheral Arterial Disease
UCC: Urgent Care Center
ICD: International Classification of Disease
RR: Relative Risk
AHA: American Heart Association
ACC: American College of Cardiology
CLTI: Chronic limb-threatening ischemia
SD: Standard Deviation
